# Enteric Pathogen Survival Varies Substantially in Irrigation Water from Belgian Lettuce Producers

**DOI:** 10.3390/ijerph111010105

**Published:** 2014-09-29

**Authors:** Inge Van Der Linden, Bart Cottyn, Mieke Uyttendaele, Nick Berkvens, Geertrui Vlaemynck, Marc Heyndrickx, Martine Maes

**Affiliations:** 1 Crop Protection—Plant Sciences Unit—Institute for Agricultural and Fisheries Research (ILVO), Burgemeester Van Gansberghelaan 96, B-9820 Merelbeke, Belgium; E-Mails: Bart.Cottyn@ilvo.vlaanderen.be (B.C.); Nick.Berkvens@ilvo.vlaanderen.be (N.B.); 2 Food Safety—Technology and Food Science Unit, Institute for Agricultural and Fisheries Research (ILVO), Brusselsesteenweg 370, B-9090 Melle, Belgium; E-Mails: Inge.Vanderlinden@ilvo.vlaanderen.be (I.V.D.L.); Geertrui.Vlaemynck@ilvo.vlaanderen.be (G.V.); Marc.Heyndrickx@ilvo.vlaanderen.be (M.H.); 3 Laboratory of Food Microbiology and Food Preservation, Department of Food Safety and Food Quality, Ghent University, Coupure Links 653, B-9000 Ghent, Belgium; E-Mail: Mieke.Uyttendaele@UGent.be; 4 Faculty of Veterinary Sciences, Department of Pathology, Bacteriology and Poultry Diseases, Ghent University, Salisburylaan 133, B-9820 Merelbeke, Belgium; 5 Laboratory of Microbiology, Department of Biochemistry and Microbiology, Faculty of Sciences, Ghent University, K.L. Ledeganckstraat 35, B-9000 Ghent, Belgium

**Keywords:** *Escherichia coli* O157:H7, *Salmonella*, enteric pathogens, irrigation water, lettuce, fresh produce

## Abstract

It is accepted that irrigation water is a potential carrier of enteric pathogens, such as *Salmonella* and *E. coli* O157:H7 and, therefore, a source for contamination of fresh produce. We tested this by comparing irrigation water samples taken from five different greenhouses in Belgium. The water samples were inoculated with four zoonotic strains, two *Salmonella* and two *E. coli* O157:H7 strains, and pathogen survival and growth in the water were monitored up till 14 days. The influence of water temperature and chemical water quality was evaluated, and the survival tests were also performed in water samples from which the resident aquatic microbiota had previously been eliminated by filter sterilization. The pathogen’s survival differed greatly in the different irrigation waters. Three water samples contained nutrients to support important growth of the pathogens, and another enabled weaker growth. However, for all, growth was only observed in the samples that did not contain the resident aquatic microbiota. In the original waters with their specific water biota, pathogen levels declined. The same survival tendencies existed in water of 4 °C and 20 °C, although always more expressed at 20 °C. Low water temperatures resulted in longer pathogen survival. Remarkably, the survival capacity of two *E. coli* 0157:H7 strains differed, while *Salmonella* Thompson and *Salmonella* Typhimurium behaved similarly. The pathogens were also transferred to detached lettuce leaves, while suspended in two of the water samples or in a buffer. The effect of the water sample on the pathogen’s fitness was also reproduced on the leaves when stored at 100% relative humidity. Inoculation of the suspension in buffer or in one of the water samples enabled epiphytic growth and survival, while the pathogen level in the other water sample decreased once loaded on the leaves. Our results show that irrigation waters from different origin may have a different capacity to transmit enteric pathogens and an important impact on the fitness of the pathogens to sustain and even grow on the leaf surface.

## 1. Introduction

*Salmonella enterica* and *Escherichia coli* O157:H7 (*E. coli* O157:H7) are the two most important bacterial pathogens associated with foodborne illness caused by the consumption of fresh produce [1]. Lettuce is the single most implicated commodity [2]. Fresh produce may become contaminated at every stage of the production process. However, before harvest, irrigation water is considered an important introduction route [3,4,5,6]. Groundwater may become contaminated by leaching of material through the soil, originating from, e.g., organic manure or feces from adjacent fields, whereas pond water may also directly become contaminated by fecal deposition [3,4,7,8,9]. These different water sources are used for irrigation by Belgian growers that produce lettuce in greenhouses. There is, however, a lack of information to what extent the risk for contamination of the plant products is comparable in different situations [10]. In the present research, the survival capacity of the pathogens in irrigation water samples from five Belgian lettuce producing sites was investigated. These waters were characterized and stored at 4 °C (±average minimum winter temperature Belgian surface water) and 20 °C (±average maximum summer temperature Belgian surface water) [11,12], with and without the addition of four enteric bacterial strains, two *Salmonella* and two *Escherichia coli* O157:H7 strains. The interaction of the pathogens with the resident aquatic biota and the influence of the chemical water quality was studied by comparing the survival of the pathogenic strains in previously filter sterilized and untreated water. Pathogen survival was compared in these water conditions, but also afterwards when transmitted to leaves, which was tested in a lab-scale experiment.

## 2. Experimental Section

### 2.1. Strains and Growth Conditions

For the survival experiments in irrigation water, four pathogen strains were used. *Salmonella* Thompson RM1987N, a spontaneous nalidixic acid-resistant mutant of *Salmonella* Thompson strain RM1987, was kindly donated by Dr. Maria Brandl (USDA-ARS, Albany, CA, USA). Strain RM1987 is a previously described clinical isolate from a patient in a cilantro-linked outbreak in California [13]. *Salmonella* Typhimurium PT 120/ad MB4880 (molecular bacteriology collection of the molecular bacteriology lab of Institute for Agricultural and Fisheries Research (ILVO)—Technology and Food Science Unit, Melle, Belgium) was isolated from overshoes at a pig farm in Belgium. *E. coli* O157:H7 MB3885 was isolated from beef carpaccio and kindly donated by the Scientific Institute for Public Health (Brussels, Belgium) and *E. coli* O157:H7 NCTC12900 by Dr. Martin Woodward (Department of Bacteriology, Veterinary Laboratories Agency(VLA) Weybridge, New Haw, Addlestone, Surrey, UK). Both *E. coli* O157:H7 isolates lack Shiga toxin genes (*stx1* and *stx2*) and were used for biosafety reasons as non-toxigenic surrogate strains for the Shiga toxin producing (STEC) serotype O157:H7. For *E. coli* O157:H7 MB3885, the absence of the *stx1* and *stx2* genes and the presence of other virulence genes, *eaeA* (intimin), *ehx* (enterohemolysin), *espP* (extracellular serine protease) and *katP* (catalase-peroxidase), were confirmed by conventional PCR, as previously described [14]. *E. coli* O157:H7 NCTC12900 originated from a verocytotoxigenic strain, which lost its ability to produce toxin. It was already used in several studies as a surrogate strain [15,16,17,18]. 

For the experiments with artificially inoculated lettuce, green fluorescent protein (GFP) transformed strains were used: *Salmonella* Thompson RM1987N [13] and *E. coli* O157:H7 MB3885 (our own constructs, plasmid pGFP, Clontech, Palo Alto, CA, USA). GFP labeled strains were used to be able to distinguish the pathogens from the natural background microbiota on the lettuce leaves, as previously described (among others [13,19,20,21]). 

All strains were taken from a glycerol frozen stock maintained at −70 °C, streaked onto a tryptone soy agar (TSA) plate (Oxoid, Basingstoke, UK) and incubated at 37 °C for 24 h. A single colony from the plate was transferred to 10 mL of tryptone soy broth (TSB) (Oxoid, Basingstoke, UK) and incubated at 37 °C for 20 h at 200 rpm. The appropriate antibiotic was added to these media when GFP labeled strains were used. This was 15 µg/mL gentamicin (G1264, Sigma-Aldrich, St. Louis, MO, USA) for *Salmonella* Thompson RM1987N GFP and 50 µg/mL ampicillin (G9518, Sigma-Aldrich) for *E. coli* O157:H7 MB3885 GFP. 

The stability of the GFP plasmid in the bacteria was examined by tracing the GFP expression, as previously described [22], but with a few modifications. The GFP labeled bacteria were inoculated into TSB broth without antibiotics. Samples of the cultures were diluted (1:1000) in fresh medium daily, incubated for 24 h, 200 rpm, 37 °C and transferred again. Plate counting on TSA plates with or without antibiotic was performed daily to quantify the functional stability of the plasmid. The fluorescence of the colonies was checked under UV light (366 nm). Non-fluorescent colonies and randomly selected fluorescent colonies were streaked onto the appropriate selective medium. This was xylose lysine desoxycholate agar (XLD) (LAB032; Lab M, Bury, UK) for *Salmonella* and cefixime-tellurite sorbitol Mac Conkey agar (CT-SMAC) (Lab 161; Lab M) for *E. coli* O157:H7. The plates were incubated at 37 °C for 24 h. 

### 2.2. Irrigation Water Samples

Irrigation water was collected (10 L samples) at four commercial greenhouses in Belgium where lettuce is grown and at the greenhouse complex of ILVO. Three ground water (GW1–3) samples and two pond water (PW1–2) samples were taken in a sterile manner.

These water samples (untreated) were stored for maximum 24 h at 4 °C before the start of the experiment. Parallel to this, subsamples were sterilized by passing through a 0.22-µm filter (Bottle Top Filters, 500-mL Capacity, MF75^™^ Series, Nalgene, Rochester, NY, USA) and stored immediately at −18 °C until used. Another set of subsamples (1 L) was analyzed by INAGRO (Rumbeke-Beitem, Belgium) and ILVO (Merelbeke, Belgium) for the following parameters: electrical conductivity (EC), pH and concentrations of Cl, SO_4_, NO_3_, NO_2_, NH_4_, Na, K, Ca, Mg, Fe, Mn and Zn. The biological oxygen demand (BOD) and chemical oxygen demand (COD) were measured for PW1 and GW1, but the values were below detection (<5 mg/L O_2_ respectively <25 mg/L O_2_). The water samples were checked for the presence of *Salmonella* and *E. coli* O157:H7. Therefore, three times 1 mL was enriched in 9 mL buffered peptone water (BPW; Oxoid, Basingstoke, UK) and incubated at 37 °C, 24 h, 200 rpm. These enrichments were then streaked onto the selective media XLD and CT-SMAC. The plates were incubated at 37 °C for 24 h and checked for presumptive colonies. Presumptive *Salmonella*-type colonies were observed in GW1 and PW1, but the colonies were negative for *Salmonella* by serological testing (DR1108, Oxoid, Basingstoke, UK). 

### 2.3. Pathogen Inoculation of Irrigation Water Samples

An overview of the different experimental conditions and used strains is shown in Table 1. Freshly grown strains were washed twice by centrifugation (13,000× g, 5 min), and the pellet was resuspended in distilled water. The optical density (595 nm) of the cultures was measured, and the appropriate amount of bacterial suspension was added to 720 mL of irrigation water in order to obtain a pathogen concentration of approximately 3.5 log CFU/mL. For each experimental condition, twelve sterile loosely capped vials (microcosms, Sigma-Aldrich, 60 mL) were filled with 20 mL of inoculated water. The vials were statically placed in the dark in a constant temperature of 20 °C or 4 °C. Static conditions were preferred, because *Salmonella enterica* and *E. coli* O157:H7 were shown to survive better under (semi)anaerobic than under aerobic conditions [23]. Uninoculated water samples (both untreated and filter sterilized) stored at 20 °C and 4 °C were used as negative controls. 

### 2.4. Quantification of Pathogen Survival in the Irrigation Water Samples

Directly after inoculation and 2, 6 and 14 days thereafter, three vials were randomly taken from each experimental condition. They were vortexed on maximum speed for 15 s, and the pathogen level was determined by plating dilutions (in 0.1% peptone) onto the corresponding selective media XLD or CT-SMAC incubated for 24 h at 37 °C and on the non-selective medium TSA incubated for 24 h at 42 °C. The choice to plate on TSA incubated at 42 °C was based on the fact that for two water samples (GW1 and PW1), plating on selective medium was impossible due to the presence of interfering non-*Salmonella* black colonies on the XLD-plates. A preliminary test had shown that the plate counts on TSA incubated at 42 °C were not significantly different from these incubated at 37 °C, while the growth of the natural background flora was strongly reduced at 42 °C. Two different controls were performed on the TSA-plates. First, there were no bacterial plate counts with the non-inoculated irrigation water samples, and secondly, randomly selected colonies grown on TSA reacted correctly in serological tests for *E. coli* 0157:H7 (DR0620, Oxoid, Basingstoke) and *Salmonella* (DR1108, Oxoid, Basingstoke). The limit of detection by plating was 0.6 log CFU/mL and achieved by plating 0.25 mL on a plate. Samples negative by plating were subjected to an enrichment step in BPW; therefore, 1 mL of the sample was added to 9 mL BPW, as described above. For the calculations, samples positive after enrichment were considered to be at the detection limit of plating (0.6 log CFU/mL), samples testing negative after enrichment were assigned a value of 0, as described by Erickson *et al.* [24]. 

**Table 1:** Overview of the 4 pathogen strains and 14 variables tested to measure pathogen survival and background bacteria in each irrigation water sample.

Water Treatment	Inoculation of the Water with					Storage Temperature of Inoculated Water
	*Salmonella* Thypimurium MB3885	*Salmonella* Thompson RM1987N	*E. coli* O157:H7 MB3885	*E. coli* O157:H7 NCTC12900	Non Inoculated	
untreated		x	x		x	4 °C
filter sterilized		x	x		x	4 °C
untreated	x	x	x	x	x	20 °C
filter sterilized		x	x		x	20 °C

### 2.5. Quantification of the Resident Heterotrophic Bacteria in the Irrigation Water Samples

For the untreated samples, the heterotrophic count was determined by plating tenfold dilutions onto water plate count agar (WPCA, 6 g/L tryptone, 15 g/L bacto agar, 3 g/L yeast extract) and incubated for 5 days at 20 °C. This was done for both the water samples inoculated with a pathogenic strain and for the negative controls. In the first case, the pathogen counts were subtracted from the WPCA counts, as described previously [25].

### 2.6. Plant Growth Conditions

Pelletized butterhead lettuce seed (*Lactuca sativa* var capitata “Alexandria RZ”) was obtained from Rijk Zwaan Distribution B.V., De Lier, The Netherlands. The plants were grown in the ILVO greenhouse in commercial potting soil (seed and cutting compost, Saniflor, Geraardsbergen, Belgium), in pots of 20-cm diameter till fully headed, mature plants (approximately 16 weeks).

### 2.7. Pathogen Inoculation of Lettuce Leaves

**Figure 1:**
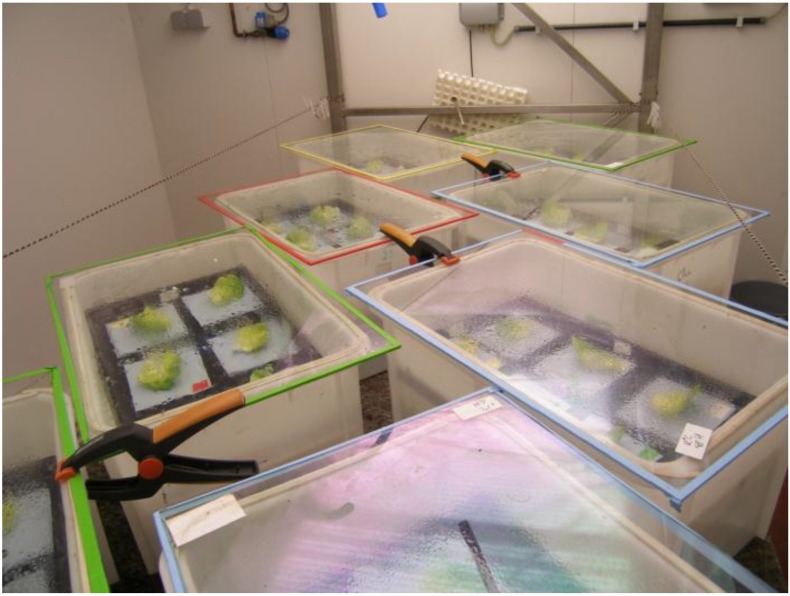
Experimental set-up of the lettuce experiment. Inoculated lettuce leaves were placed on trays. The trays were placed in plastic boxes with 100% RH in a growth chamber with a 14/10 h day/night regime at 20 °C/12 °C.

Bacterial inocula were prepared in five hundred mL of Groundwater Sample 3 (GW3), Pond Water Sample 2 (PW2) or phosphate-saline buffer (PBS) (50 mM, pH 7.4), to which GFP labelled *Salmonella* Thompson or *E. coli* O157:H7 MB3885 (in the same concentrations) were added. These suspensions were then immediately applied on plant leaves. Young inner leaves of mature lettuce crops were cut approximately 1 cm above the soil surface. For each test combination, 9 detached leaves were dipped for 3 seconds in the appropriate suspension, allowed to drip off. The initial contamination levels were ±3.5 log CFU/g leaf (see also Section 2.8). The leaves were then placed in trays with paper towel (random design), allowed to dry (30 min) in the biosafety cabinet and subsequently transferred into plastic boxes that had a 10-cm layer of water in the bottom. The boxes were closed with glass plates to reach 100% relative humidity (Figure 1) and placed in a growth chamber with a 14/10 h day/night regime at 20 °C/12 °C. Relative humidity and temperature in the boxes were logged every 5 min with an EL-USB-2 data logger (Lascar Electronics, Salisbury, UK). Pathogen levels on the leaves were followed for three days. 

### 2.8. Quantification of Pathogen Survival on the Lettuce Leaves

Thirty minutes (Day 0) and 1 and 3 days after inoculation, three leaves of each test combination were randomly selected, individually placed in a sterile extraction bag with a filter (Bioreba, Reinach, Switzerland) and weighed. After addition of PBS with Tween 20 (0.05% v/v) at a 1/1 (w/v) ratio, the leaves were ground for 15 s at maximum speed with a Homex 6 (Bioreba), which generated a homogenous mixture. Ten-fold dilutions (in 0.1% peptone) of the extracts were plated on TSA supplemented with the appropriate plasmid encoded antibiotic. The plates were incubated at 37 °C for 24 h, and the fluorescent colonies were counted under UV light (366 nm).

### 2.9. Statistical Analysis

The pathogen survival experiments in irrigation water were performed one time; three vials were analyzed at each time point for each investigated condition. The influence of the plating medium was analyzed using a full factorial negative binomial regression with day and plating medium integrated as factors in the regression model for each water sample, strain, filtering treatment and temperature combination. The influence of the different treatments on the survival of the pathogens in the irrigation water was analyzed by means of a full factorial negative binomial regression with day, temperature and filtering treatment integrated as factors in the regression model for each water sample and strain combination. The difference in survival between the different strains was analyzed using a full factorial negative binomial regression with day and strain as factors for the untreated water samples stored at 20 °C. Each analysis with the negative binomial regression models started with a saturated model, and interactions and non-significant main factors were sequentially dropped at a significance level of 0.05. The most parsimonious model was used when analyzing the data. Negative binomial regression was performed using the GENMOD procedure in SAS 9.4 (SAS Institute Inc., Cary, NC, USA). The experiments with lettuce leaves were performed twice with three lettuce leave samples on different days and with different lettuce crops. The results were analyzed by day. A non-parametric Kruskal-Wallis test with pairwise comparison and taking the necessary Bonferroni corrections into account was performed (IBM SPSS Statistics 19, Somers, NY, USA).

## 3. Results

### 3.1. Pathogen’s Survival in Irrigation Water: Influence of Water Temperature and Resident Aquatic Microbiota

**Figure 2:**
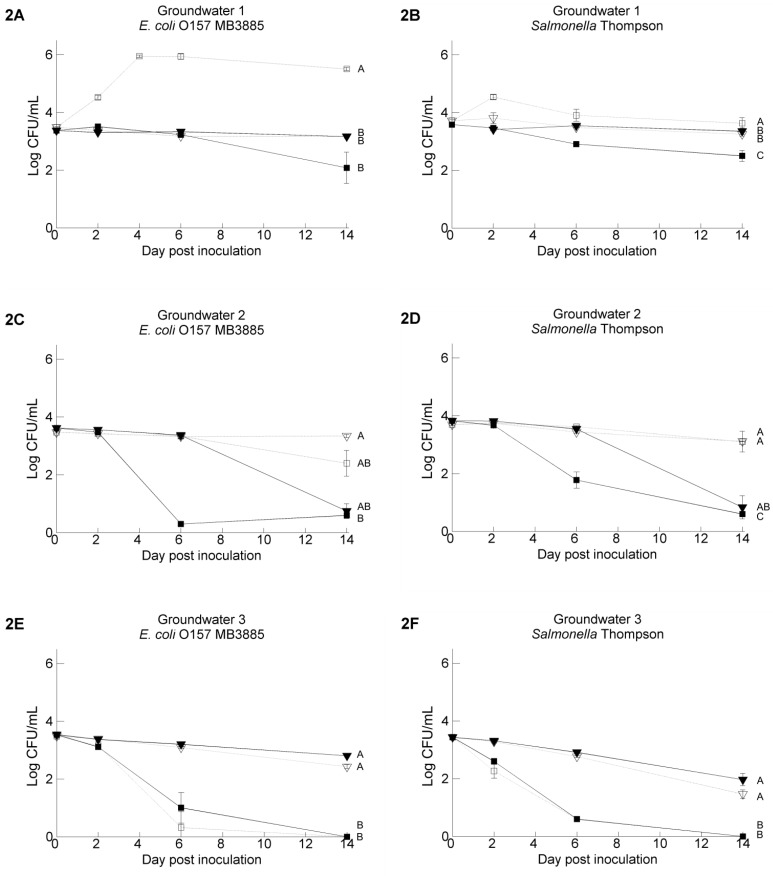
Survival of *E. coli* O157:H7 MB3885 (**left**) and *Salmonella* Thompson RM1987N (**right**) in three ground water samples with the following treatments: untreated water samples stored at 4 °C (full line, ▼), filter sterilized water samples stored at 4 °C (dashed line, ▽), untreated water samples stored at 20 °C (full line, ■), filter sterilized water samples stored at 20 °C (dashed line, □). The data show the mean of three analyzed vials and are calculated from the log transformed values of the pathogen population size. Error bars indicate standard deviations. Different letters indicate significant difference (*p* < 0.05) between means according to negative binomial regression.

**Figure 3:**
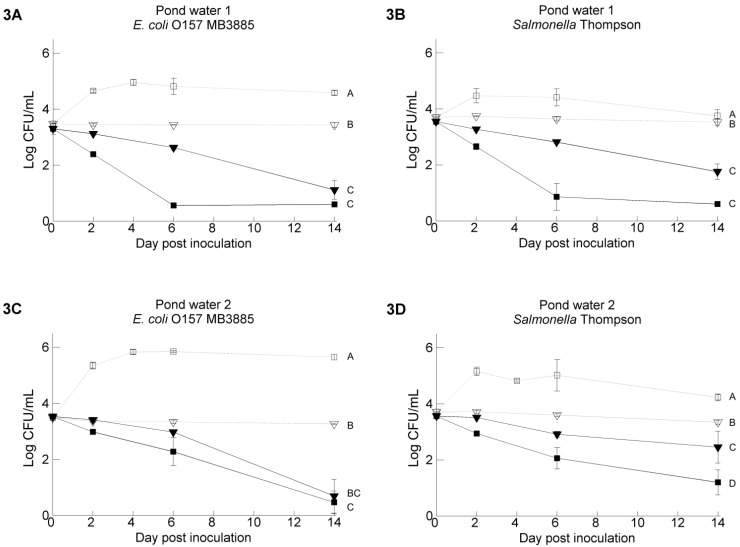
Survival of *E. coli* O157:H7 MB3885 (**left**) and *Salmonella* Thompson RM1987N (**right**) in two pond water samples with the following treatments: untreated water samples stored at 4 °C (full line, ▼), filter sterilized water samples stored at 4 °C (dashed line, ▽), untreated water samples stored at 20 °C (full line, ￭), filter sterilized water samples stored at 20 °C (dashed line, □). The data show the mean of three analyzed vials and are calculated from the log transformed values of the pathogen population size. Error bars indicate standard deviations. Different letters indicate significant difference (*p* < 0.05) between means according to negative binomial regression.

The chemical characteristics of the water samples are presented in Table 2. Figure 2A–F and Figure 3A–D show the survival of *Salmonella* Thompson (ST) and *E. coli* O157:H7 MB3885 (EC) in the five irrigation water samples. The results from the TSA platings at 42 °C are shown. In general, higher populations of pathogens were recovered from the TSA platings compared with the respective selective media (CT-SMAC and XLD). This effect was more often observed in water samples stored at 4 °C (14/15) in comparison with at 20 °C (5/15) (data not shown). Except for GW3, higher pathogen counts were observed at 20 °C in filter sterilized water in comparison with untreated water, and these differences were significant for GW1, PW1 and PW2 (for GW1, PW1 and PW2, EC and ST, *p* < 0.0001). The biggest differences between filter sterilization treatment or not were observed in PW1; in this water sample, both pathogens survived significantly better at 4 and 20 °C (ST 4 °C, ST 20 °C and EC 20 °C, *p* < 0.0001; EC 4 °C, *p* < 0.001) in filter sterilized water in comparison with the untreated samples stored at the same temperature. The highest pathogen counts were detected in water samples that were filter sterilized and stored at 20 °C, as well. Moreover, in three of these water samples, both pathogens were able to grow within the first six days after inoculation (up to 6.03 log/mL) (GW1, PW1 and PW2). This was not noticed at 4 °C. Both pathogens survived in general better in untreated water kept at 4 °C instead of 20 °C; growth was not observed. 

**Table 2:** Chemical water characteristics of the five irrigation water samples.

Water Sample	pH-H_2_O	EC	${NO}_{3}^{-}$	${NH}_{4}^{-}$	SO_4_	Cl	Fe	Mn	Mg	Ca	K	Na	Cu	Zn	NO_2_
		µS/cm	mg/L	mg/L	mg/L	mg/L	mg/L	mg/L	mg/L	mg/L	mg/L	mg/L	µg/L	mg/L	mg/L
GW1 ^a^	7.45	710	0.383	<0.22	78.64	51.2	0.52	0.15	15.60	94.30	19.00	29.40	<0.01	<0.01	<0.12
GW2 ^a^	6.99	1413.0	198.5	<0.22	340.9	48.8	0.08	0.06	43.50	219.90	58.85	38.00	0.03	0.73	<0.12
GW3 ^a^	7.56	698.0	0.9	<0.3	134.4	42.4	0.02	<0.01	15.00	102.61	8.25	12.72	<0.01	0.79	<0.3
PW1 ^b^	6.91	106	0.278	<0.22	7.28	7.6	0.14	0.06	2.37	8.77	6.75	4.18	0.01	<0.01	<0.12
PW2 ^b^	7.71	651.0	5.2	<0.22	165.5	46.3	0.02	0.02	14.80	82.65	13.20	29.70	<0.01	0.01	<0.12

Notes: ^**a**^ GW = groundwater; **^b^** PW = pond water.

### 3.2. Pathogen’s Survival in Irrigation Water: Comparisons between Water Samples and Different Strains of Each Pathogen

**Figure 4:**
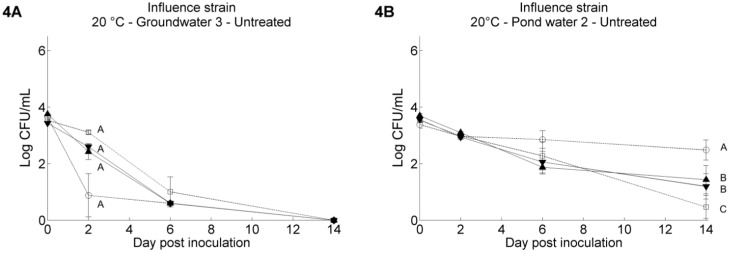
Survival of *Salmonella* Typhimurium MB4880 (▲), *Salmonella* Thompson RM1987N (▼), *E. coli* O157:H7 MB3885 (□) and *E. coli* O157:H7 NCTC12900 (○) in untreated irrigation water stored at 20 °C. Pathogen suspensions in (**A**) groundwater 3; (**B**) pond water 2. The data show the mean of three analyzed vials and are calculated from the log transformed values of the pathogen population size. Error bars indicate standard deviations. Different letters indicate significant difference (*p* < 0.05) between means according to negative binomial regression.

The pathogen’s survival was significantly different between the different irrigation water samples (*p* < 0.001). This was especially clear at 20 °C, and less at 4 °C. Besides that, irrigation water GW3 showed a very different pathogen survival profile. In general, similar survival trends were observed for *Salmonella* Thompson and *E. coli* O157:H7 under the same experimental conditions, although some differences could be seen: *E. coli* O157:H7 MB3885 survived significantly better than *Salmonella* Thompson RM1987N in some test conditions, such as in sterilized GW1 (*p* < 0.001, Figure 2A,B) and sterilized PW2 at 20 °C (*p* < 0.001, Figure 3C,D) and in untreated GW3 at 4 °C (0.001 < *p* < 0.01, Figure 2E,F). To test whether the observed differences were strain specific or species specific, the survival of two more strains, *Salmonella* Typhimurium MB4880 and *E. coli* O157:H7 NCTC12900, was tested in the untreated water samples stored at 20 °C. The results for GW3 and PW1 are shown in Figure 4; the results for the other water samples can be found in the Supplemental Material, Figure S1. The survival of the two *Salmonella* strains was in general not significantly different from each other, whereas for the two *E. coli* O157:H7 strains, a difference in pathogen level up to 2.0 log CFU/mL existed when residing in the PW2 water at Day 14 after inoculation. In four of the five water samples, *E. coli* O157:H7 NTCT12900 survived less well than *E. coli* O157:H7 MB3885, whereas the opposite was observed in GW3. 

### 3.3. The Resident Background of Heterotrophic Microorganisms in the Irrigation Water Samples

**Figure 5:**
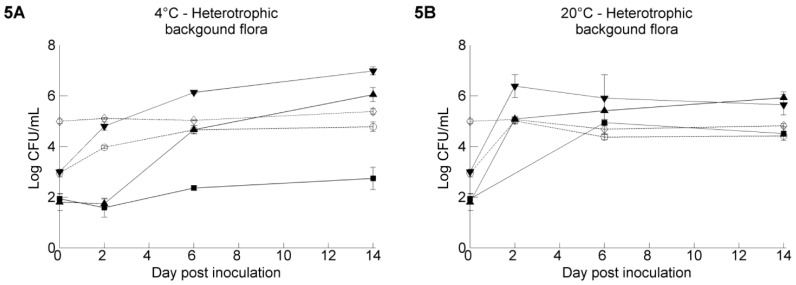
Plate counts of heterotrophic background microbiota residing in the five irrigation water samples (Ground Water 1 (▲), Ground Water 2 (▼), Ground Water 3 (￭), Pond Water 1 (◊), Pond Water 2 (○)) stored at 4 °C **(A)** and 20 °C **(B)**. The data show the mean of three analyzed vials and are calculated from the log transformed values of the heterotrophic background population size. Error bars indicate standard deviations.

The population dynamics of the heterotrophic background microbiota are shown in Figure 5. The initial counts varied from 1.81 ± 0.30 log CFU/mL to 5.00 ± 0.08 log CFU/mL between the different irrigation water samples and increased during the 14-day storage experiment. Levels up to 6.98 ± 0.16 log CFU/mL were observed in GW2 at 4 °C at Day 14. The increase was faster, but not higher at 20 °C (within two days) than at 4 °C. The counts were not significantly different from the level of resident bacteria in the non-inoculated irrigation water samples. No statistically significant correlation could be found between the growth/die off rate of the pathogens and the heterotrophic background microbiota for any of the water samples.

### 3.4. Pathogen Survival on Butterhead Lettuce Leaves: Influence of the Inoculum Carrier

**Figure 6:**
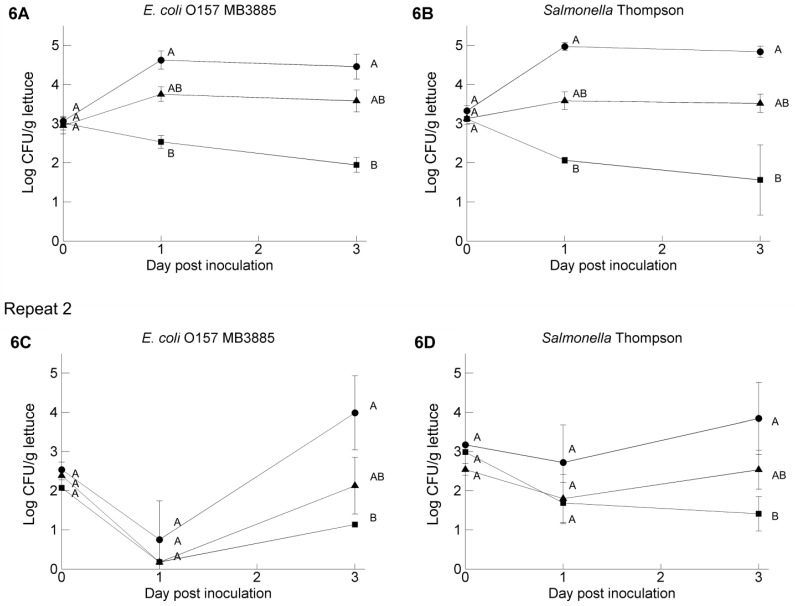
Levels of GFP labeled *E. coli* O157:H7 MB3885 (**A**,**C**) and GFP labeled *Salmonella* Thompson RM1987N (**B**,**D**) from two independent repeats, recovered from lettuce leaves at three time points after inoculation with the pathogens suspended in phosphate buffer-saline (●), pond water 2 (▲) or ground water 3 (￭). Data presented are calculated from the log transformed values of the pathogen population size on three leaf samples and expressed per gram of leaf tissue. Error bars indicate standard deviations. At each day, different letters indicate significant difference (*p* < 0.05) between means of pathogen level according to Kruskal-Wallis non-parametric test.

The preliminary test showed that the GFP plasmid remained present in both strains; green fluorescence was detected in more than 99% of the colonies up to the end of the test (Day 10). When *Salmonella* and *E. coli* O157:H7 were suspended in PBS, GW3 or PW2 as the carrier to inoculate lettuce leaves, significantly different concentrations of these pathogens were recovered from the leaves (Figure 6) (0.01 < *p* < 0.05). The highest concentrations were recuperated from leaves inoculated with suspensions in PBS and then followed by the variant in PW2. In contrast, when inoculated in GW3 water, the amount of pathogens on the leaves declined. *E. coli* O157:H7 MB3885 and *Salmonella* Thompson behaved in a similar way. 

## 4. Discussion

This study was conducted to evaluate the extent to which enteric pathogens survive in irrigation water from different origin and quality and to have a first estimate of the implication for contamination of fresh green produce. We used butterhead lettuce as the test plant. Lettuce cultivation in a greenhouse is common in Belgium, and irrigation water reservoirs were sampled on five different production sites. In the first part of our study, the survival of *E. coli* O157:H7 and *Salmonella* was followed in artificially inoculated irrigation water samples from different Belgian lettuce production sites. The influence of temperature, the presence of resident aquatic microbiota, chemical water quality or pathogen strain was tested. A schematic summary and tentative interpretation of our results performed at 20 °C is shown in Table 3.

For natural waters, it is known that temperature has a significant effect on the survival of enteric pathogens [7], with better survival at lower temperatures [26,27]. This was also seen in the present experiment in the untreated water samples. However, in the filter sterilized water samples, the opposite was observed. In three irrigation water samples, even growth of the pathogen could be noticed at 20 °C. These results are in accordance with Vital *et al.* [28,29], who have shown that growth of *E. coli* O157:H7 can be observed in sterilized natural freshwater at low carbon concentrations when the initial inoculation concentration is not higher than the so-called “carrying capacity” of the water. This may also indicate that sterilized irrigation water could be a risk factor when the contamination event occurs after the sterilization treatment. By comparing the pathogen survival in both filter sterilized and untreated water samples, the influence of the presence of the heterotrophic background microbiota and the chemical water quality could be determined. In general, the survival of *E. coli* O157:H7 and *Salmonella* was significantly better in the sterilized water samples. This indicates that for these samples, competition with the resident aquatic microbiota may be responsible for the decline of the pathogens in the untreated water samples. 

Bacterial competition is a very complex process for which the current state of knowledge of the contributing factors (such as nutrient dynamics, concentrations of competing species) is very limited for natural waters [28]. These issues were also addressed in a simulation model for the decline of *E. coli* O157:H7 in various natural substrates [30]. Furthermore, also the presence of protozoa may have influenced the survival of enteric pathogens in irrigation water. Ravva *et al.* has shown that selected types of protozoa preferentially engulf specific isolates of *E. coli* O157:H7, while some protozoa engulf the pathogen in the presence of specific nutrients [31,32]. This may be one of the factors that can explain the pronounced differences that were observed for the two *E. coli* O157:H7 strains, but the hypothesis could not be confirmed, as protozoal counts were not performed. 

**Table 3:** Schematic overview of the results of survival of *Salmonella* and *E. coli* O157:H7 in 5 irrigation water samples which were previously filter sterilized or not and subsequently stored at 20 °C.

Water Sample	Initial Level Resident Heterotrophic Bacteria	Endpoint Pathogen Level		Tentative Interpretation		
		in presence of aquatic biota (in untreated water)	in absence of aquatic biota (in filter sterilized water)	combination of competitive survival and growth and nutrient availability	risk of pathogen transfer when using non-sterilized irrigation water	risk of pathogen transfer in case of pathogen contamination of sterilized water
GW1	low	high	very high	-very low pathogen suppression (low bacterial background load)	high	very high
				-nutrients for pathogen growth available		
GW2	medium	low	high	-important pathogen suppression by resident aquatic biota	low	high
				-limited nutrients for pathogen growth available		
				-high Zn level		
GW3	low	low	low	-important pathogen suppression but not by the resident aquatic biota	low	low
				-the pathogen does not survive in this water, although the bacterial background does		
				-high Zn level		
PW1	high	low	very high	-important pathogen suppression by resident aquatic biota	low	very high
				-nutrients for pathogen growth available		
PW2	medium	medium	very high	-weaker pathogen suppression by resident aquatic biota	medium	very high
				-nutrients for pathogen growth available		

In two water samples (GW2 and GW3), the pathogen behavior in the untreated samples could not (only) be explained by competition with the resident aquatic microbiota. In GW2 the survival of the pathogen was significantly better in the sterilized water sample, but no growth could be observed; and in GW3, no difference in survival was seen between the untreated and filter sterilized water. This may indicate that for these water samples, also the chemical water quality may have had a significant influence on the survival of the pathogen. One of the factors that may explain these results is the fact that water samples were characterized by a higher Zn concentration. For GW3, we could show that this high concentration originated most likely from galvanized irrigation pipes and the irrigation storage tank, as the water that was sampled directly from the borehole reservoir was characterized by a much lower Zn concentration. When a survival experiment was conducted with this borehole water sample, growth of the pathogen could be observed in the sterilized water sample (data not shown). Toxic effects of zinc on bacteria have been reported [33]. It was shown that a concentration of 0.25 mg/L has a direct toxic effect (20 min) on *E. coli* and that a longer exposure time significantly increases the sensitivity of *E. coli* to metal pollutants [33]. In a similar survival experiment as ours, Avery *et al.* [34] found a significant negative correlation between the mean log CFU *E. coli* O157:H7 and log Zn concentration. In GW2, very high levels of zinc were found, as well, yet differences in filtering did change the pathogen survival. This may indicate that the Zn-complex in the two water samples was not the same. Furthermore, other toxic chemical elements may have been present in GW3, which were not analyzed, or synergistic effects between different chemical elements could have occurred [33]. 

These results (influence competition microbiota, chemical composition) show high similarities with the survival of enteric pathogens in various other natural substrates, such as manure and slurry [35].

In the second part of our study, two of the artificially inoculated water samples were used to introduce the pathogens onto butterhead lettuce leaves to evaluate their subsequent survival. These results were compared with PBS as the inoculum carrier, together with sterile distilled water and other standard sterile buffers commonly used. Only a few studies have used irrigation water as the inoculum carrier [36,37], probably because this makes it more difficult to repeat the experiment in exactly the same conditions. To our knowledge, only Theofel and Harris [38] and Choi *et al.* [39] have investigated the influence of the inoculum carrier on the subsequent survival of the pathogen on leafy greens. Theofel and Harris did not find significant differences when Milli Q water, 0.1% peptone water or pond water was used as an inoculum carrier to deliver *E. coli* O157:H7 to fresh-cut lettuce that was subsequently stored at either 5 or 20 °C. Although the average inoculum level was comparable with our study, the inoculation method was different. We used a 300-times higher inoculum volume with a hundred-times lower inoculum level. Furthermore, the pathogen was more evenly distributed on the leaves with our dip inoculation method in comparison with their spot inoculation. This may explain why we were able to observe a significant effect of the inoculum carrier, although also other experimental factors could have played a role (different strain, lettuce variety, storage temperature, *etc.*). Choi *et al.* were able to see differences in *E. coli* O157:H7 survival on lettuce when sterile distilled water or peptone water as inoculum carrier was used, with better survival with peptone water. They used 100 µL inoculum for each leaf and suggested that the organic matter in peptone water protects *E. coli* O157:H7 from environmental stresses and/or provides nutrients to support colonization in an environment with 100% relative humidity. In our test, the pathogen’s survival on the plant was comparably better when introduced in PBS, but with the current experimental design, it was not possible to explain whether this effect was due to the absence of resident background microbiota in the sterile buffer, the chemical composition of the irrigation water samples or a combination of these factors.

We chose to perform the experiment with detached butterhead leaves with the same leaf age in conditions of high relative humidity in order to keep the variation as low as possible [25,38]. This is important when small differences in the survival of enteric pathogens on fresh produce need to be investigated. However, even under these standardized conditions, the survival between the different repeats were significantly different, although the same trends could always be observed: better survival on the lettuce when introduced with PBS as the inoculum carrier, less survival when introduced with GW3. The tested high humidity conditions (100%) are, however, not typical of preharvest conditions. Under drier conditions, it is therefore likely that populations of both groups would have declined, such as described previously, and it would have been difficult to discern an effect of the irrigation water quality on the overall pathogen survival [40,41].

In order to test the hypotheses that were put forward in this study and to investigate the impact of the irrigation water quality on enteric pathogen’s survival on lettuce under commercial growth conditions in detail, further research is required. In such follow up studies, a higher number of water samples should be investigated, and the experiments should be repeated in time, in order to take the chemical and microbiological variability of the water into account. Furthermore, it should be interesting to characterize the nutrient availability of the water samples in more detail, e.g., by measuring the dissolved organic content (DOC) per unit microbial biomass [9,21], and to determine the low pathogen levels more accurately, e.g., by applying the most probable number method (MPN-method).

## 5. Conclusions

Our study confirms that the survival of *Salmonella* and *E. coli* O157:H7 may vary between different irrigation water samples. The individual pathogen’s fitness for leaf colonization at high relative humidity seems to be influenced by the quality of the irrigation water.

## Supplementary Files

Supplementary File 1

## References

[1] Sivapalasingam S., Friedman C.R., Cohen L., Tauxe R.V. (2004). Fresh produce: A growing cause of outbreaks of foodborne illness in the United States, 1973 through 1997. J. Food Prot..

[2] Rangel J.M., Sparling P.H., Crowe C., Griffin P.M., Swerdlow D.L. (2005). Epidemiology of *Escherichia coli* O157:H7 outbreaks, United States, 1982–200. Emerg. Infect. Dis..

[3] Nygård K., Lassen J., Vold L., Andersson Y., Fisher I., Löfdahl S., Threlfall J., Luzzi I., Peters T., Hampton M. (2008). Outbreak of *Salmonella* Thompson infections linked to imported rucola lettuce. Foodborne Pathog. Dis..

[4] Söderström A., Österberg P., Lindqvist A., Jönsson B., Lindberg A., Blide Ulander S., Welinder-Olsson C., Löfdahl S., Kaijser B., de Jong B. (2008). A large *Escherichia coli* O157 outbreak in Sweden associated with locally produced lettuce. Foodborne Pathog. Dis..

[5] Gu G., Hu J., Cevallos-Cevallos J.M., Richardson S.M., Bartz J.A., van Bruggen A.H.C. (2011). Internal colonization of *Salmonella enterica* serovar Typhimurium in tomato plants. PLoS One.

[6] Cevallos-Cevallos J.M., Danyluk M.D., Gu G., Vallad G.E., van Bruggen A.H. (2012). Dispersal of *Salmonella* Typhimurium by rain splash onto tomato plants. J. Food Prot..

[7] Steele M., Odumeru J. (2004). Irrigation water as source of foodborne pathogens on fruit and vegetables. J. Food Prot..

[8] Semenov A.V., van Overbeek L., van Bruggen A.H. (2009). Percolation and survival of *E. coli* O157:H7 and *Salmonella enterica* serovar Typhimurium in soil amended with contaminated dairy manure or slurry. Appl. Environ. Microbiol..

[9] Gu G., Luo Z., Cevallos-Cevallos J.M., Adams P., Vellidis G., Wright A., van Bruggen A.H. (2012). Factors affecting the occurrence of *Escherichia coli* O157 contamination in irrigation ponds on produce farms in the Suwannee River watershed. Can. J. Microbiol..

[10] Holvoet K. (2014). Bacterial Safety of Lettuce in Primary Production and Fresh-Cut Processing Industry.

[11] Anibas C., Buis K., Verhoeven R., Meire P., Batelaan O. (2011). A simple thermal mapping method for seasonal spatial patterns of groundwater-surface water interaction. J. Hydrol..

[12] Van Vliet M., Zwolsman J. (2008). Impact of summer droughts on the water quality of the Meuse river. J. Hydrol..

[13] Brandl M.T., Miller W.G., Bates A.H., Mandrell R.E. (2005). Production of autoinducer 2 in *Salmonella enterica* serovar Thompson contributes to its fitness in chickens but not on cilantro leaf surfaces. Appl. Environ. Microbiol..

[14] Verstraete K., de Reu K., van Weyenberg S., Piérard D., de Zutter L., Herman L., Robyn J., Heyndrickx M. (2013). Genetic characteristics of Shiga toxin-producing *E. coli* O157, O26, O103, O111 and O145 isolates from humans, food, and cattle in Belgium. Epidemiol. Infect..

[15] Dibb-Fuller M.P., Best A., Stagg D.A., Cooley W.A., Woodward M.J. (2001). An *in vitro* model for studying the interaction of *Escherichia coli* O157:H7 and other enteropathogens with bovine primary cell cultures. J. Med. Microbiol..

[16] Skandamis P.N., Nychas G.J.E. (2000). Development and evaluation of a model predicting the survival of *Escherichia coli* O157:H7 NCTC12900 in homemade eggplant salad at various temperatures, pHs, and oregano essential oil concentrations. Appl. Environ. Microbiol..

[17] Vande Walle K., Atef Yekta M., Verdonck F., de Zutter L., Cox E. (2011). Rectal inoculation of sheep with *E. coli* O157:H7 results in persistent infection in the absence of a protective immune response. Vet. Microbiol..

[18] Woodward M.J., Best A., Sprigings K.A., Pearson G.R., Skuse A.M., Wales A., Hayes C.M., Roe J.M., Low J.C., la Ragione R.M. (2003). Non-toxigenic *Escherichia coli* O157:H7 strain NCTC12900 causes attaching-effacing lesions and *eae*-dependent persistence in weaned sheep. Int. J. Med. Microbiol..

[19] Semenov A.M., Kuprianov A.A., van Bruggen A.H. (2010). Transfer of enteric pathogens to successive habitats as part of microbial cycles. Microb. Ecol..

[20] Franz E., Visser A.A., van Diepeningen A.D., Klerks M.M., Termorshuizen A.J., van Bruggen A.H.C. (2007). Quantification of contamination of lettuce by GFP-expressing *E. coli* O157:H7 and *Salmonella enterica* serovar Typhimurium. Food Microbiol..

[21] Franz E., Klerks M.A., de Vos O.J., Termorshuizen A.J., van Bruggen A.H.C. (2007). Prevalence of Shiga toxin-producing Escherichia coli stx(1), stx(2), eaeA, and rfbE genes and survival of *E. coli* O157:H7 in manure from organic and low-input conventional dairy farms. Appl. Environ. Microbiol..

[22] Lapidot A., Yaron S. (2009). Transfer of *Salmonella enterica* serovar Typhimurium from contaminated irrigation water to parsley is dependent on curli and cellulose, the biofilm matrix components. J. Food Prot..

[23] Semenov A.V., van Overbeek L., Termorshuizen A.J., van Bruggen A.H. (2011). Influence of aerobic and anaerobic conditions on survival of *Escherichia coli* O157:H7 and *Salmonella enterica* serovar Typhimurium in Luria-Bertani broth, farm-yard manure and slurry. J. Environ. Manag..

[24] Erickson M.C., Liao J., Payton A.S., Riley D.G., Webb C.C., Davey L.E., Kimbrel S., Ma L., Zhang G., Flitcroft I. (2010). Preharvest internalization of *Escherichia coli* O157:H7 into lettuce leaves, as affected by insect and physical damage. J. Food Prot..

[25] Brandl M.T., Amundson R. (2008). Leaf age as a risk factor in contamination of lettuce with *Escherichia coli* O157:H7 and *Salmonella enterica*. Appl. Environ. Microbiol..

[26] Rice E.W., Johnson C.H., Wild D.K., Reasoner D.J. (1992). Survival of *Escherichia coli* O157:H7 in drinking water associated with a waterborne disease outbreak of hemorrhagic colitis. Lett. Appl. Microbiol..

[27] Wang G., Doyle M.P. (1998). Survival of enterohemorrhagic *Escherichia coli* O157:H7 in water. J. Food Prot..

[28] Vital M., Hammes F., Egli T. (2008). *Escherichia coli* O157 can grow in natural freshwater at low carbon concentrations. Environ. Microbiol..

[29] Vital M., Stucki D., Egli T., Hammes F. (2010). Evaluating the growth potential of pathogenic bacteria in water. Appl. Environ. Microbiol..

[30] Semenov A.V., Franz E., van Bruggen A.H. (2010). COLIWAVE a simulation model for survival of *E. coli* O157:H7 in dairy manure and manure-amended soil. Ecol. Model..

[31] Ravva S.V., Sarreal C.Z., Mandrell R.E. (2010). Identification of protozoa in dairy lagoon wastewater that consume *E. coli* O157:H7 preferentially. PLoS One.

[32] Ravva S., Sarreal C., Mandrell R., Michael Davidson J.F.P., Ryser E.T., Sofos J.N. (2013). Intra and Inter-Strain Differences in Fitness of E. coli O157:H7 to Protozoan Predation and Survival in Soil.

[33] Preston S., Coad N., Townend J., Killham K., Paton G.I. (2000). Biosensing the acute toxicity of metal interactions: Are they additive, synergistic, or antagonistic?. Environ. Toxicol. Chem..

[34] Avery L.M., Williams A.P., Killham K., Jones D.L. (2008). Survival of *E. coli* O157:H7 in waters from lakes, rivers, puddles and animal-drinking troughs. Sci. Total Environ..

[35] Semenov A.V., van Bruggen A.H.C., van Overbeek L., Termorshuizen A.J., Semenov A.M. (2007). Influence of temperature fluctuations on *E. coli* O157:H7 and *Salmonella enterica* serovar Typhimurium in cow manure. FEMS Microbiol. Ecol..

[36] Barker-Reid F., Harapas D., Engleitner S., Kreidl S., Holmes R., Faggian R. (2009). Persistence of *E. coli* on injured iceberg lettuce in the field, overhead irrigated with contaminated water. J. Food Prot..

[37] Solomon E.B., Pang H.J., Matthews K.R. (2003). Persistence of *E. coli* O157:H7 on lettuce plants following spray irrigation with contaminated water. J. Food Prot..

[38] Theofel C.G., Harris L.J. (2009). Impact of preinoculation culture conditions on the behavior of *E. coli* O157:H7 inoculated onto romaine lettuce (*Lactuca sativa*) plants and cut leaf surfaces. J. Food Prot..

[39] Choi S., Bang J., Kim H., Beuchat L.R., Ryu J.H. (2011). Survival and colonization of *E. coli* O157:H7 on spinach leaves as affected by inoculum level and carrier, temperature and relative humidity. J. Appl. Microbiol..

[40] Brandl M.T., Mandrell R.E. (2002). Fitness of *Salmonella enterica* serovar Thompson in the cilantro phyllosphere. Appl. Environ. Microbiol..

[41] Van der Linden I., Cottyn B., Uyttendaele M., Vlaemynck G., Heyndrickx M., Maes M. (2013). Survival of enteric pathogens during butterhead lettuce growth: Crop stage, leaf age, and irrigation. Foodborne Pathog. Dis..

