# Supplementary material for: Enteric Pathogen Survival Varies Substantially in Irrigation Water from Belgian Lettuce Producers
Source: Int J Environ Res Public Health. 2014 Sep 29;11(10):10105–24. doi: 10.3390/ijerph111010105 (PMC4210970; doi:10.3390/ijerph111010105)
Supplement: Supplementary File 1 [file ijerph-11-10105-s001.pdf]

Supplementary Information

Enteric Pathogen Survival Varies Substantially in Irrigation Water from Belgian Lettuce Producers

---

Figure S1. Survival of *Salmonella* Typhimurium MB4880 (▴), *Salmonella* Thompson RM1987N (▾), *E. coli* O157:H7 MB3885 (□) and *E. coli* O157:H7 NCTC12900 (○) in untreated irrigation water stored at 20 ℃. Pathogen suspensions (**A**) Groundwater 1, (**B**) Groundwater 2, (**C**) Pond Water 1. The data show the mean of triplicate samples and are calculated from the log-transformed values of the pathogen population size. Error bars indicate standard deviations. Different letters indicate significant difference according to negative binomial regression ($p < 0.05$).

S1A

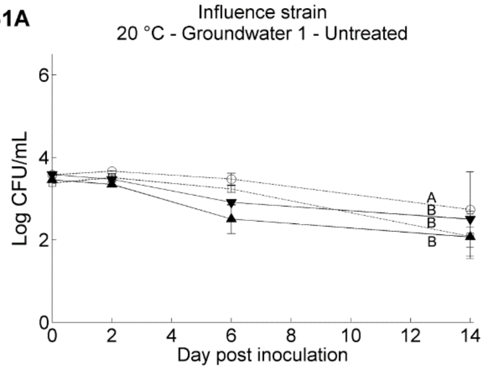

S1B

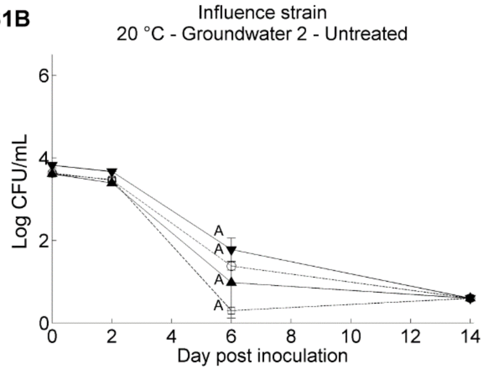

S1C

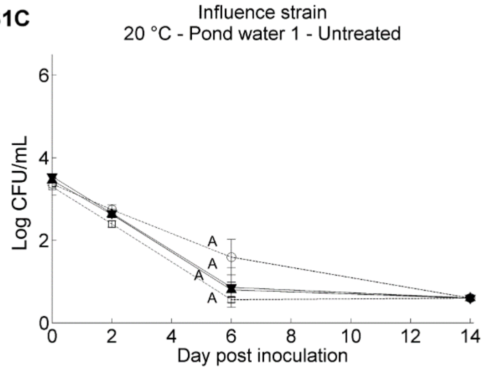

© 2014 by the authors; licensee MDPI, Basel, Switzerland. This article is an open access article distributed under the terms and conditions of the Creative Commons Attribution license (http://creativecommons.org/licenses/by/4.0/).
